# Spondyloarthritis and the Human Leukocyte Antigen (HLA)-B^*^27 Connection

**DOI:** 10.3389/fimmu.2021.601518

**Published:** 2021-03-08

**Authors:** Chengappa G. Kavadichanda, Jie Geng, Sree Nethra Bulusu, Vir Singh Negi, Malini Raghavan

**Affiliations:** ^1^Department of Clinical Immunology, Jawaharlal Institute of Postgraduate Medical Education and Research, Puducherry, India; ^2^Department of Microbiology and Immunology, University of Michigan Medical School, Ann Arbor, MI, United States

**Keywords:** HLA-B^*^27, spondyloarthritis, ER stress, free heavy chain, IL-23/IL-17 axis, ERAP1

## Abstract

Heritability of Spondyloarthritis (SpA) is highlighted by several familial studies and a high association with the presence of human leukocyte antigen (HLA)-B^*^27. Though it has been over four decades since the association of HLA-B^*^27 with SpA was first determined, the pathophysiological roles played by specific HLA-B^*^27 allotypes are not fully understood. Popular hypotheses include the presentation of arthritogenic peptides, triggering of endoplasmic reticulum (ER) stress by misfolded HLA-B^*^27, and the interaction between free heavy chains or heavy chain homodimers of HLA-B^*^27 and immune receptors to drive IL-17 responses. Several non-HLA susceptibility loci have also been identified for SpA, including endoplasmic reticulum aminopeptidases (ERAP) and those related to the IL-23/IL-17 axes. In this review, we summarize clinical aspects of SpA including known characteristics of gut inflammation, enthesitis and new bone formation and the existing models for understanding the association of HLA-B^*^27 with disease pathogenesis. We also examine newer insights into the biology of HLA class I (HLA-I) proteins and their implications for expanding our understanding of HLA-B^*^27 contributions to SpA pathogenesis.

## Introduction

**Figure 1:**
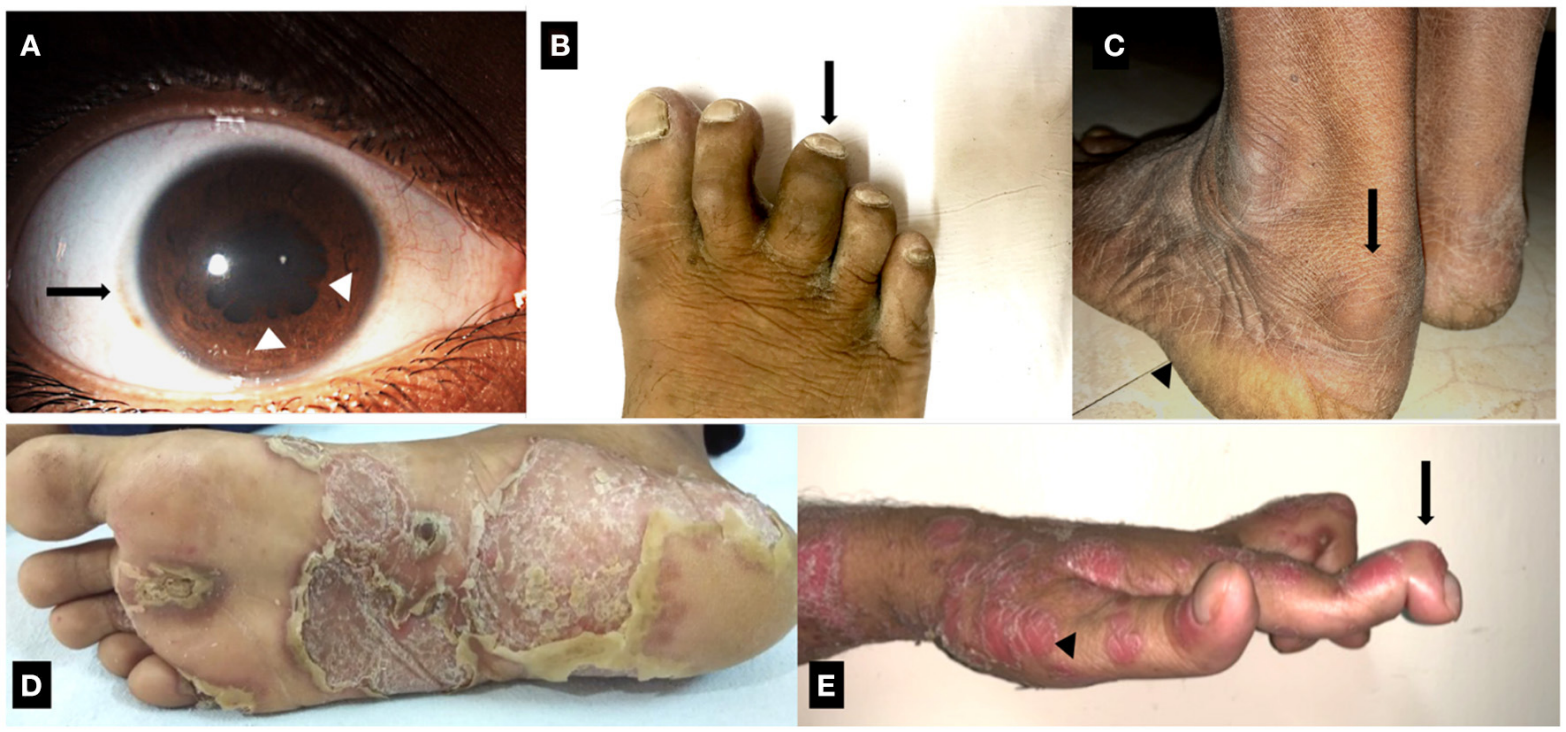
Clinical manifestations of Spondyloarthritis. **(A)** Resolving recurrent acute anterior uveitis of right eye in a case of ankylosing spondylitis. Solid arrow: mild circum-corneal congestion. Arrowhead: posterior synechiae. **(B)** Dactylitis of third toe of the right foot in a case of Psoriatic arthritis. **(C)** Enthesitis involving the Achilles tendon in a case of ankylosing spondylitis. Solid arrow: retrocalcaneal bursitis, which co-occurs with Achilles enthesitis. Arrowhead: site of plantar fasciitis. **(D)** Keratoderma blennorrhagica involving the sole of a patient with Reactive arthritis. **(E)** Psoriasis (arrowhead) with deforming peripheral arthritis of hand joints. Solid arrow: arthritis and deformity of distal interphalangeal joint.

Spondyloarthritis (SpA) is a group of seronegative arthritides, which includes ankylosing spondylitis (AS), psoriatic arthritis (PsA), reactive arthritis (ReA), undifferentiated SpA and enteropathy related arthritis (EA) (1). The global prevalence of SpA ranges from 0.2 to 1.61% in the general population. The numbers depend on the geographic area, the study population, data sources and the case definition used to classify SpA, which has evolved considerably over the years (2). The above subtypes of SpA share several phenotypic characteristics (Figure 1), which include inflammatory lesions in the axial and peripheral joints, enthesitis (inflammation at the insertion sites of tendons and ligaments into the bone), uveitis (inflammation in the eye) and enteritis (inflammation in the small intestine), in varying combinations and frequencies. Clinically, individuals with SpA present with low back ache, alternating gluteal pain and stiffness of the spine, all of which worsen with rest and improve upon exercise. Along with these axial symptoms, individuals with SpA also have inflamed peripheral joints and entheseal sites. The skeletal manifestations are often associated with several extra-articular features in the eye, skin and gut, depending on the subtype of SpA. The features start off insidiously and progress chronically in most of the subtypes of SpA except in ReA. ReA presents with an acute onset of ankle or knee inflammation along with dactylitis (combined inflammation of the joint and soft tissues in fingers or toes), enthesitis, conjunctivitis and skin lesions like keratoderma blennorhagicum (mucous-laden skin lesions) (Figure 1) and circinate balanitis (skin inflammation around the glans penis) (3). PsA is an SpA phenotype which occurs in individuals with psoriatic skin lesions. It is characterized by a variable combination of sacroiliitis (inflammation of the sacroiliac joints), which is usually asymmetrical, oligo (affecting 2–4 joints) to polyarthritis (affecting > 4 joints), enthesitis, dactylitis (Figure 1) and chronic anterior or posterior uveitis (4). AS is considered as the prototype SpA and it presents with bilateral symmetric sacroiliitis, acute anterior uveitis, peripheral arthritis and enthesitis (5). SpA can have varying degrees of bowel inflammation ranging from microscopic asymptomatic colitis to overt inflammatory bowel disease (IBD) in 7–10% of AS and PsA (6). Besides this, 3% of patients with IBD have AS, 10% have subclinical sacroiliac joint involvement and 30% of the cases have SpA-like musculoskeletal symptoms (7).

The skeletal manifestations progress from inflammatory lesions to a combination of erosive, destructive and proliferative pathology. The proliferative pathology, characterized by new bone formation, progresses partly independent of the inflammatory process. In particular, the axial skeletal involvement progresses to cause severe disability as a result of irreversible fusion of vertebral bodies and formation of marginal syndesmophytes (calcification and new bone formation in ligaments). This results in a stiff spine referred to as the “bamboo spine” even in patients whose inflammation is fairly controlled. The reason for this progression is still not elucidated and remains as an important unanswered question in the management of SpA.

In inflammatory arthritic diseases, such as Rheumatoid Arthritis (RA), the commonly used and most effective immunosuppressive agents are glucocorticoids (GC) and conventional synthetic disease-modifying anti-rheumatic drugs (csDMARDS). For unknown reasons, these drugs have negligible benefit in SpA. On the other hand, non-steroidal anti-inflammatory drugs (NSAIDs) have excellent efficacy in relieving pain and spinal stiffness in SpA. Besides NSAIDs, monoclonal antibodies against tumor necrosis factor alpha (TNFα) and IL-17 are the current standard of care in SpA (8). Even though these biological agents reduce inflammation, it remains unclear if these drugs can reduce new bone formation. The response to the cytokine-targeted therapies are not uniform and there are a substantial number of patients who do not respond to either of these drugs. It is unclear who will respond to each drug and there is no convincing basis for one drug choice over the other. It is likely that numerous factors including genetic factors have a role in determining the differences in the onset, progress and response to treatment in SpA.

As discussed below, the association between SpA and HLA-B^*^27 is one of the strongest known associations between an HLA allele and disease. Several studies have highlighted the importance of this association for diagnosis, predicting disease phenotype and prognosis of SpA. However, our current understanding of the pathophysiologic pathways linking HLA-B^*^27 and SpA is still incomplete. In this review, we summarize the clinical associations between HLA-B^*^27 in patients with SpA and among their family members. We also examine the role of HLA-B^*^27 in enthesitis, new bone formation and gut pathology encountered in SpA. Finally, we elaborate on the unique properties of HLA-B^*^27 and highlight the newer findings related to HLA-B^*^27 biology which may partially explain the relevance of HLA-B^*^27 to SpA.

## HLA-B^*^27 and Disease Phenotype of SpA

The occurrence of AS in a first degree relative (FDR) or other family members of the proband was known much before the identification of HLA-B^*^27-SpA association (9). The strength of association between HLA-B^*^27 and various SpA categories are variable (Table 1). The presence of HLA-B^*^27 results in the onset of AS symptoms at an early age (10, 11). The presence of HLA-B^*^27 also determines the distribution of inflammation across various organs in SpA. Uveitis, hip arthritis and sacroiliitis are more common among HLA-B^*^27^+^ individuals (12), whereas the presence of HLA-B^*^27 in AS is negatively associated with peripheral arthritis and dactylitis (11).

**Table 1:** Association of HLA-B*27 and various classes of Spondyloarthritis.

**Diseases**	HLA-B*27 frequency	**Comments**
Ankylosing Spondylitis (AS)	75–90% (12)	Prototypical SpA, which is classified based on the modified New York criteria, heavily banks on X-ray changes of sacroiliac joints.
Non-Radiographic Spondyloarthritis (Nr-SpA)	75–90% (11,13)	This subclass was earlier classified as undifferentiated SpA.
Reactive arthritis (ReA)	30–60% (3)	Few reports have linked HLA-B*27 with chronicity of ReA.
Psoriatic Arthritis (PsA)	20–50% (18)	HLA-B*27 positive patients with psoriatic arthritis have higher incidence of enthesitis, dactylitis and symmetric sacroiliitis.
Enteropathy/Inflammatory bowel diseases related arthritis (EA/ IBD-SpA)	10–40% (7)	HLA-B*27 is likely to increase the likelihood of having axSpA features in those with inflammatory bowel disease.
Enthesitis related arthritis (ERA)	50–80% (21)	The juvenile counterpart of spondyloarthritis. They have predominant oligo arthritis in the beginning and a few patients gradually progress to have axial symptoms in the early adulthood.
Undifferentiated peripheral Spondyloarthritis (USpA)	25–70%	This class is now limited to enteropathy related arthritis without overt features of IBD or PsA before the onset of psoriatic skin lesions.

Recent reports studying the broader classification of SpA, which include the non-radiographic phenotype, have found higher disease activity and worse functional scores in the B^*^27^−^ patients (11, 13) suggesting that HLA-B^*^27^−^ patients are likely to have worse disease at baseline, possibly as a result of delay in diagnosis. The presence of HLA-B^*^27 is likely to result in a prolonged course of illness as reflected by radiographic damage. Studies involving MRI imaging of sacroiliac joints have shown higher structural damage and inflammatory edema among HLA-B^*^27^+^ patients (14). Moreover, when followed over several years, the progress in structural damage of sacroiliac joints and the propensity to develop marginal symmetric syndesmophytes along the vertebral column are higher among the HLA-B^*^27^+^ individuals (15, 16).

HLA-B^*^27 is associated with onset of arthritis in individuals who developed psoriasis after 40 years of age (17). The presence of HLA-B^*^27 is also associated with bilateral sacroiliitis which otherwise is asymmetrical and unilateral in PsA (18). Inflammation in the sacroiliac joints detected by MRI is higher in HLA-B^*^27^+^ PsA patients, which is similar to that seen in AS (18). In EA, presence of HLA-B^*^27 is associated with higher incidence of lower limb arthritis (19).

SpA in children and adolescents are currently classified either as enthesitis related arthritis (ERA) and juvenile psoriatic arthritis (jPsA) or as juvenile Spondyloarthritis (jSpA). The presence of HLA-B^*^27 among these individuals predisposes to development of skeletal deformities (20). Unlike their adult counterparts, patients with ERA have lower limb-predominant oligoarthritis, and late onset of axial involvement (21).

Overall, the data associate HLA-B^*^27 with uveitis and early onset disease, with radiologically severe axial manifestation in AS. The non-AS SpA group is predisposed to develop axial and peripheral in the presence of HLA-B^*^27. Among the jSpA, HLA-B^*^27 appears to be associated with a poorer prognosis.

### Gut Inflammation in SpA

SpA is characterized by inflammation in mainly four tissues, either in isolation or in combination. The gut, entheses, anterior chamber of the eye, and axial skeleton including the sacroiliac, intervertebral, costotransverse and facet joints are the tissues predominantly affected. The gut and entheses are proposed as the sites where inflammation is initiated (22, 23). Evidence garnered from histo/immuno-pathological studies, animal studies, and *in vitro* cell-based studies involving these tissues have furthered our understanding of SpA and are summarized here.

The epithelial barrier along with the mucosa-associated lymphoid tissue and the microbiota of an individual controls the delicate equilibrium between immune tolerance and activation. The gut involvement in SpA can range from overt IBD, to subclinical microscopic colitis, to dysbiosis in almost all patients with SpA (6, 24). Early experiments demonstrated that HLA-B^*^27 transgenic mice failed to develop SpA phenotype when reared in a germ-free environment (25). Reports have also shown that HLA-B^*^27 may alter the composition of the gut microbiota. *Bacteroides vulgatus*, for instance, is abundantly present in the HLA-B^*^27 transgenic Lewis lines as compared to the wild-type controls (26). Similarly, studies on gut biopsies of humans with AS have demonstrated higher colonies of certain bacterial communities (*Lachnospiraceae, Rikenellaceae, Porphyromonadaceae*, and *Bacteroidaceae*) (27) and a strong positive correlation of genus *Dialister* (28) and *Ruminococcus gnavus* (27, 29) with disease activity. The latter study also showed significant differences in microbiota composition between HLA-B^*^27^+^ and HLA-B^*^27^−^ siblings of AS (29), further suggesting a role for B^*^27 in determining the composition of microbiome in humans. These findings indicate that HLA-B^*^27 may have a role in altering immune responses by modifying the gut microbiome. The development of gut inflammation in HLA-B^*^27 transgenic rats can be altered by administering oral antibiotics. This treatment also reduces IL-1α and CCL2 levels and the number of Lin^−^CD172a^+^CD43^low^ monocytes, subsets shown to have osteoclastogenic potential (30). A recent study investigating the role of metabolites in the gut of HLA-B^*^27 transgenic rat found that HLA-B^*^27 expression alters the intestinal metabolome of rats even before the onset of SpA symptoms. Moreover, administration of microbial metabolite propionate could attenuate development of SpA phenotype in these rats (31). It is, however, still not clear if the dysbiosis is due to inflammation or vice-versa.

Dysbiosis and presence of invasive bacteria in gut of AS alters the gut epithelial and gut vascular barrier along with dysregulated zonulin and tight junction expression. The leakiness due to altered tight junction in the gut results in increased levels of zonulin and bacterial products such as lipopolysaccharide (LPS), LPS-binding protein (BP), and intestinal fatty acid-BP in the serum of patients with AS (32) which can influence the differentiation of circulating monocytes.

The IL-23 and IL-17 pathways are linked to many autoimmune and auto-inflammatory diseases including AS (33–35). Genetic studies have suggested links between IL-23R, autoimmunity (33), gut inflammation (36) and AS (34), and some studies have reported increased IL-23 expression in AS (37, 38). Overexpression of IL-23 in the gut is thought to be a marker of intestinal inflammation, with Paneth cells (PC) being a major source of IL-23 (37). Studies with HLA-B^*^27 transgenic rats have demonstrated links between HLA-B^*^27 misfolding, the unfolded protein response (UPR) and IL-23 hyper-production (39). Other studies have indicated that misfolding of HLA-B^*^27 induces autophagy in the gut and downstream regulation of the production of IL-23 in AS (40). IL-23 can then activate Th17 cells, ILC3 cells, mucosal-associated invariant T (MAIT) cells, and γδ T cells involved in type 3 immunity (41). Overall, it seems that HLA-B^*^27, dysbiosis and activation of the IL-23/IL-17 axis at the gut are primal for inducing inflammation at remote sites including the joints and enthesis. However, the presence of all the SpA features including gut inflammation in HLA-B^*^27^−^ SpA suggests that the precise role of B^*^27 in regulating the IL-23/IL-17 axis needs to be further explored. Other possible links between HLA-B^*^27 and IL-23/IL-17 axis will be further discussed below.

### Enthesitis in SpA

The enthesis is a fibrocartilaginous part of a ligament or tendon that inserts to the bone surface. Besides acting as a structure anchoring the muscle, tendon or capsule to the bone, the enthesis is a complex organ that efficiently transmits mechanical forces from muscles to bones across joints. Chronic inflammation of the entheseal complex (enthesitis) is a common clinical occurrence in SpA. In patients with SpA, enthesitis in the lower limbs (Achilles tendon and plantar fascia) is more common than that in upper limbs, probably due to the microtrauma burden at the former sites. In the TNF^Δ*ARE*^ mouse, which lack on-off regulation of TNF biosynthesis (42), enthesitis and new bone formation were promoted only upon inducing biomechanical stress (43), suggesting a key role for mechanical stress in the pathogenesis of SpA. Indeed, many sites subject to high mechanical stress, including the aortic root, the ciliary body of the eye, skin extensor surfaces, and the lung apex are target sites in the SpA group of diseases (44).

IL-23 seems to play an important role in precipitating enthesitis and resulting bone remodeling in SpA. Sherlock et al. found that entheseal sites of mice expressing IL-23 contained a population of CD3^+^CD4^−^CD8^−^IL-23R^+^, RAR-related orphan receptor γt (ROR-γt)^+^ T cells. They further demonstrated that IL-23 overexpression was sufficient to precipitate enthesitis which predated the structural changes in joints, induced by prolonged inflammation. The IL-23 mediated pathology was dependent on the presence of CD3^+^CD4^−^CD8^−^IL-23R^+^ROR-γt^+^ T cells and was independent of the presence of CD4^+^ T cells including the Th17 cells. IL-23 induced expression of TNFα, IL-17 and IL-22 by the newly described subset of entheseal T cells (45). Furthermore, IL-22 was the dominant effector cytokine driving bone remodeling at the site of entheseal inflammation (45).

The clinical trial results of anti-IL-23 and anti-IL-17 in SpA indicate different effects. While anti-IL-17 agents had good outcomes in patients with axSpA (46, 47), anti-IL-23 agents failed to show benefits over placebo (48, 49). On the other hand, IL-23 inhibitors were beneficial in treating enthesitis in PsA (50), but the efficacy in improving arthritis and axial symptoms was not as impressive as those with anti-TNFs (46). These results have led to several untested hypotheses about the role of IL-23 in SpA. IL-23 may be an initiator for IL-17A and TNFα release, and axSpA may represent a chronic and mature form with a predominant IL-17-related phenotype. The other hypothesis is that the pathways driving the axial inflammation and peripheral inflammation have distinct etiologies and that IL-23 predominantly drives the peripheral SpA phenotype.

Another important finding in SpA is the detection of innate-like lymphocyte 3 (ILC 3) cells and γδ T cells at entheseal sites. Human entheses are shown to contain ILC3, based on expression of RORγt and IL-23R, and these cells induced IL-17A transcripts upon stimulation with IL-23 and IL-1β (51). These experiments were carried out on entheseal tissues of healthy individuals who had spinal surgeries and whose HLA-B^*^27 status was not known. It remains to be elucidated whether and how HLA-B^*^27 and chronic inflammation will alter this response. γδ T cells are also known to be a major source of IL-17 and TNFα. Experiments using mouse models and human enthesis have demonstrated that subsets of entheseal γδ T-cells may be maintained as self-renewing tissue resident cells. These cells upregulate IL-17A production in an IL-23-independent (52) or dependent (53) manner when activated, making them important players in local inflammation. Even though an earlier study in humans showed higher levels of IL-23R^+^ γδ T cells in the peripheral blood of HLA-B^*^27^+^ AS patients (54), their role as entheseal resident cells in humans is yet to be established. Biomechanical or unknown infective stressors are likely the triggers for enthesitis. These stressors possibly trigger IL-23-mediated responses at the entheseal site, which is self-perpetuating in some cases leading to widespread inflammation. More precise information about the entheseal resident cell populations, their interactions with HLA-B^*^27 and their role in injury and healing may provide us with better insights into the pathophysiology of enthesitis in SpA.

### New Bone Proliferation

In SpA, the process of intense inflammation and repair is accompanied by formation of bony spicules from the underlying trabecular bone. New bone formation is not limited to HLA-B^*^27-mediated disease. Non-HLA-B^*^27 transgenic animal models like the Dilute, Brown and non-Agouti (DBA) 1 (55), Ankylosing enthesopathy (ANKENT) mice (56), and mouse models with overexpression of IL-23 show evidence of new bone formation, while TNF overexpressing models like the TNF^Δ*ARE*^ mice do not show osteoproliferation (43, 45). Data from long term follow-up of patients treated with anti-TNF agents have not been conclusive about prevention of osteoproliferation (57). These findings suggest that there are factors beyond inflammation that are likely to result in new bone formation. The presence of HLA-B^*^27 appears to increase the severity and magnitude of osteoproliferation in AS (15, 16) and PsA (18). Inflammation, neoangiogenesis, and new bone formation are visualized in clinical practice even in asymptomatic entheseal sites of SpA patients using ultrasound power doppler, MRI, and radiographs, and are HLA-B^*^27-dependent (58). The process of new bone formation in AS appears to be a result of trans-differentiation followed by ossification of cartilage and direct bone formation (59). The common understanding is that new bone formation is facilitated by bone morphogenic proteins (BMPs) and Wnt proteins. In DBA/1 mice, new bone formation was driven by BMP signaling, which was inhibited by Noggin, a BMP antagonist (60). Studies in humans using immunohistochemistry of synovial tissue showed higher expression of BMP-2 and BMP-6 in inflamed SpA tissue, but not non-inflamed tissue (61). These genes were up-regulated in fibroblast-like synoviocytes by IL-1β and TNFα, which are important cytokines in SpA pathogenesis. Another study in AS patients showed abnormal secretion of Noggin and BMP2, by mesenchymal stem cells (MSC), causing an imbalance that is suggested to induce abnormal osteogenic differentiation (62). The IL-17 and IL-22 accumulated at the enthesis during acute inflammation may also facilitate the proliferation, migration and osteogenic differentiation of MSCs (63).

As noted above, Sherlock et al. attributed new bone formation to be IL-23 and IL-22 driven (45). Some recent studies have shed light on a potential direct pathway linking HLA-B^*^27 and osteoproliferation. Expression of HLA-B^*^27:04 and B^*^27:05 but not B^*^07:02 along with human beta2-microglobulin (hβ2m) in *Drosophila* resulted in the loss of cross veins in the wings. This phenotype resulted from a dominant-negative effect on the BMP receptor saxophone, and elevated phosphorylation of a *Drosophila* receptor mediated Smad. The human saxophone ortholog ALK2 and HLA-B^*^27 were shown to interact in lymphoblastoid cells from AS patients. Active ALK2 inhibits BMP signaling via phosphorylation of Smads. HLA-B^*^27-mediated inhibition of ALK2 is suggested to result in uncontrolled activation of TGF-β/BMP signaling pathway, resulting in osteoproliferation (64). Yet another study using MSCs from the spinal entheseal sites of AS patients demonstrated that mineralization of MSCs was increased by the HLA-B^*^27–mediated spliced X-box–binding protein 1 (sXBP1)/retinoic acid receptor-β (RARB)/tissue-nonspecific alkaline phosphatase (TNAP) axis (65). Further elucidating the relationship between HLA-B^*^27 and the ALK2 and TNAP axes will be important for a better understanding the pathogenesis of new bone formation in SpA.

## Role of HLA-B^*^27 in SpA Pathogenesis

**Figure 2:**
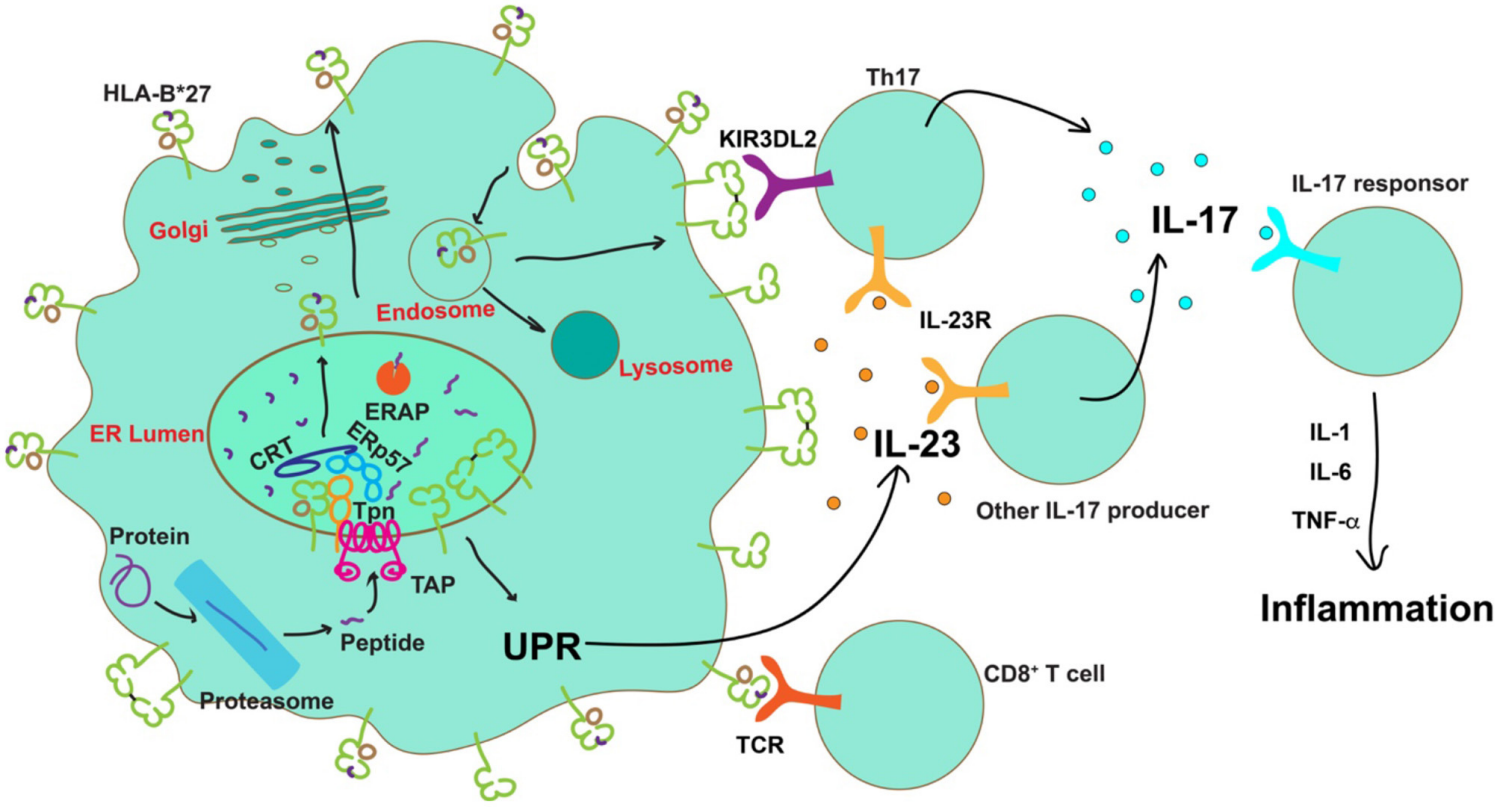
Possible roles of HLA-B*27 in the pathogenesis of AS. After transport into the ER by the transporter associated with antigen processing (TAP), peptides are assembled onto nascent MHC class I molecules by the peptide loading complex (PLC), comprising TAP, tapasin (Tpn) calreticulin (CRT) and ERp57, and further trimmed by ERAP. The presence of certain ERAP haplotypes results in a significantly decreased number of peptides optimal for HLA-B*27, leading to accumulation of misfolded proteins in the ER or expression of sub-optimally or neoantigen-loaded HLA-B*27 on the cell surface. Neoantigen loaded HLA-B*27 on the cell surface could induce activation of CD8^+^ T cells. Protein misfolding in the ER can cause ER stress and activation of the unfolded protein response (UPR) to induce IL-23 secretion. IL-23 further activates the IL-23/IL-17 axis. On the other hand, free heavy chains or disulfide bond-linked homodimers of HLA-B*27 can be formed and expressed on the cell surface during HLA-B*27 recycling via the endocytic pathway. Engagement of these aberrant species by KIR3DL2 on the surface of Th17 cells is known to enhance their survival, proliferation, and IL-17 expression. IL-17 can promote the release of proinflammatory cytokines to establish inflammation.

HLA-I molecules are highly polymorphic, comprising a heavy chain, a light chain [β2-microglobulin (β2m)] and a short peptide. The connections between HLA-B^*^27 and SpA must correlate with their distinct properties from the other HLA-I allotypes. HLA-B^*^27 allotypes have a preference for peptides with an arginine at the P2 position, as do some other allotypes. A genetic study argues that the strongest association with SpA was a unique asparagine at residue 97 in HLA-B^*^27, which lies on the floor of the peptide-binding groove and determines the specificity for C-terminal residue of the peptide (66). In addition, quantitative repertoire differences at the peptide C-terminus were also identified between SpA-linked and non-SpA-linked HLA-B^*^27 allotypes (67). If peptide specificity is the basis for connection between HLA-B^*^27 and SpA, C-terminal residues might be critical. On the other hand, different from many other HLA-I allotypes, HLA-B^*^27 allotypes have a free cysteine at the residue 67, which was shown to promote the formation of misfolded versions, including free heavy chains (FHC) and intermolecular disulfide bond-mediated heavy chain homodimers (B^*^27_2_) (68). It was reported that the non-SpA-linked allotype HLA-B^*^27:09 show reduced HLA-B^*^27 dimer formation, compared with SpA-associated HLA-B^*^27 allotype HLA-B^*^27:05 (69). However, a more recent study examining the abilities of eight HLA-B^*^27 allotypes to form homodimers suggests that homodimer formation does not reflect different associations with SpA (70). In line with these features of HLA-B^*^27, there are three main hypotheses of the role played by HLA-B^*^27 in SpA pathogenesis, as illustrated in Figure 2.

### Arthritogenic Peptide Theory: HLA–B^*^27 Presents Specific Antigens to CD8^+^ T Cells

Given the strong associations between SpA and HLA-B^*^27 and the major role of HLA-I in antigen presentation to CD8^+^ T cells, SpA was initially considered as a disorder of adaptive immunity. The “arthritogenic peptide theory” proposed that, under certain conditions, some HLA-B^*^27 allotypes present self-peptides to specific CD8^+^ T cells, causing damage to self-tissues. An early study showed that higher levels of CD8^+^ T cells in the entheses of SpA patients (71). A recent study also showed that CD8^+^ T cells are recruited to synovial fluid from the blood of AS patients (72). Additionally, self-peptide-responsive CD8^+^ T cells have been identified in the peripheral blood mononuclear cells (PBMCs) or synovial fluid of some AS patients (73, 74). However, the arthritogenic peptide theory is challenged by the findings in transgenic rat models. Onset and severity of SpA manifestations were not affected in CD8α-deficient rat model (75), indicating that CD8^+^ T cells are not essential for the development of SpA, nudging investigators to look beyond the classical functions of MHC-I molecules to better understand disease precipitation.

### HLA-B^*^27 Induces ER Stress

Based on recent models, SpA is suggested to be more related to innate rather than adaptive immune responses. As described above, specific properties of HLA-B^*^27 are known to cause the accumulation of unfolded and misfolded HLA-B^*^27 in the ER, which are suggested to potentially activate UPR. UPR is a set of intracellular pathways that signal the presence of ER stress upon sensing of misfolded proteins. Sustained over-activation of the UPR has been implicated in multiple inflammatory diseases. The possible relationship between HLA-B^*^27 misfolding, UPR and SpA pathogenesis was first investigated in an HLA-B^*^27 transgenic rat models. The rat line 33-3 expressing 55 copies of HLA-B^*^27:05 and 66 copies of hβ2m had predominant gut disease. High copies of B^*^27 in this model lead to protein misfolding in the ER resulting in UPR activation in bone marrow-derived macrophages and in the gut. In contrast, similar experiments with HLA-B^*^07, which is from a different supertype with different folding properties, did not show HLA-I misfolding and UPR activation (76). HLA-B^*^27 misfolding-activated UPR in bone marrow-derived macrophages augmented IL-23 expression induced by LPS. Increase of UPR in the colon of HLA-B^*^27 transgenic rats was associated with increased activation of the IL-23/IL-17 axis (39). These findings provide possible links between HLA-B^*^27 misfolding and dysregulation of immune responses in SpA patients. However, inconsistent with the HLA-B^*^27 misfolding-UPR theory, increasing β2m copy numbers (which promotes HLA-B^*^27 folding in the ER and mitigates UPR) enhanced the severity of AS symptoms in HLA-B^*^27 transgenic rats (77). These results argue against the crucial role of HLA-B^*^27 misfolding in the ER for SpA development.

Studies from human subjects are also inconclusive. While in one study, elevated ER stress and UPR activation was observed in monocytes/macrophages from peripheral blood and synovial fluid of AS patients (78), ER stress or UPR activation was not seen in other studies with peripheral blood monocyte-derived macrophages from HLA-B^*^27^+^ AS patients (79, 80). UPR activation was also not observed in the gut of individuals with AS (40). Of note, HLA-B^*^27 has recently been shown to lower the threshold for UPR induction, and UPR might be activated in AS patients by a combination of HLA-B^*^27 misfolding and infection by certain bacteria (81). In addition, there appears to be a relationship between autophagy and IL-23 expression in the ileum of HLA-B^*^27^+^ AS patients (40).

### Cell Surface HLA–B^*^27 FHC and B^*^27_2_ Regulate Immune Responses

HLA-B^*^27 FHC or B^*^27_2_ are expressed on the cell surface, and these aberrant forms of HLA-B^*^27 could be generated in the endosomes during the constitutive recycling of the cell surface HLA-B^*^27 molecules (82). These non-conventional HLA-B^*^27 conformers are suggested to be more readily detected in HLA-B^*^27^+^ AS patients than HLA-B^*^27^+^ healthy donors (83). Inhibiting HLA-B^*^27_2_ with the monoclonal antibody HD6 prevented IL-17 secretion by PBMCs from HLA-B^*^27^+^ SpA patients (83). These findings suggest cell surface aberrant HLA-B^*^27 conformers might be induced in SpA and play important roles in SpA pathogenesis.

Leukocyte immunoglobulin (Ig)-like receptors (LILR), such as LILRA1, LILRB2 (84), and LILRB5 (85) are shown to recognize HLA-B^*^27 FHC or B^*^27_2_. B^*^27_2_ also binds to the paired immunoglobulin-like receptors (PIR) in rodents (86), which share homology in their ligand binding domains to the LILR families in humans. How the interaction between B^*^27_2_ and LILR/PIR connects to enhanced inflammation is not yet well-established. On the other hand, HLA-B^*^27 FHC and B^*^27_2_ were reported to bind the killer cell immunoglobulin-like receptor (KIR)-KIR3DL2 more strongly than other ligands (87). Engagement of KIR3DL2 by B^*^27_2_ promotes the survival of KIR3DL2^+^ NK cells, and peripheral blood NK cells from SpA patients showed higher cytotoxicity than those from RA patients and healthy controls (88). In addition to NK cells, KIR3DL2 is also expressed on CD4^+^ T cells. The engagement of KIR3DL2 by HLA-B^*^27 FHC and B^*^27_2_ was shown to increase the survival and proliferation of Th17 cells from AS patients (89), consistent with the finding that Th17 cells are enriched in the peripheral blood and synovial fluid of patients with early axSpA (90). Importantly, if the interaction between aberrant HLA-B^*^27 conformers and a specific receptor is critical for AS pathogenesis, this receptor is likely to be conserved across species, given that HLA-B^*^27 induces AS or AS-like disease in both human and rodent animals.

### Role of ERAP

Although there is a strong association between HLA-B^*^27 and AS, a majority of the HLA-B^*^27 individuals never develop disease, implying that HLA-B^*^27 is not the only risk factor. In addition to cytokine pathways discussed previously, GWAS studies have shown that the ER aminopeptidase ERAP1 has strong genetic association with AS, in addition to HLA-B^*^27 (34). ERAP1 and ERAP2 are ER-localized aminopeptidases, which trim ER peptides at the N-terminus to produce peptides of optimal length for HLA-I binding and presentation (91). ERAP1 association is only significant in specific subsets of individuals who are HLA-B^*^27^+^ (92) or HLA-B^*^40^+^ (66), implying that AS development depends on the combined effect of HLA-B and ERAP1. The association between ERAP1 and HLA-B^*^27 has been widely studied. As an ER-localized aminopeptidase, ERAP1 influences both the quality and quantity of peptides available for HLA-B^*^27 in the ER, and thus, influences the peptide repertoire of HLA-B^*^27 expressed on the cell surface. ERAP1 single nucleotide polymorphisms (SNP), which are distinctly associated with AS, are proposed to cause differences in peptide cleavage efficiency and substrate selectivity, further emphasizing the importance of peptide loading of HLA-B^*^27 in AS pathogenesis. The most significant ERAP polymorphisms associated with AS include rs30187, that encodes the K528R variant, and rs27044, that encodes the Q730E variant (33, 93). In general, K528 is associated with more efficient cleavage, while Q730 is associated with increased preference for shorter peptides. However, the effect of each residue on peptide cleavage efficiency is not absolute, but rather is dependent on ERAP1 polymorphisms at other residues (94, 95). Early studies have shown that ERAP1 allotypes with high cleavage efficiency are risk factors for AS development, while allotypes with diminished activity are protective. Follow-up studies from Reeves et al. suggest that, based on their cleavage efficiencies, ERAP1 variants can be roughly divided into hyperactive (over-trimming), normal and hypoactive (inefficient trimming) allotypes. Hyperactive allotypes are always detrimental and hypoactive allotypes are detrimental when two are co-expressed, and these scenarios are uniquely present in AS patients (95). The study also found that ERAP1 allotype combinations from AS patients are less efficient in promoting HLA-B^*^27:05 expression than those from non-AS controls, suggesting abnormal peptide cleavage (either too strong or too weak) and inefficient peptide loading of HLA-B^*^27 are responsible for AS onset (95). However, this theory was challenged by analysis of ERAP1 SNP haplotypes from a larger cohort of AS patients and RA controls, which argues that ERAP1 allele combinations in AS patients reported by Reeves et al. are pretty rare, and that most ERAP1 allele combinations from AS patients are shared with RA controls and do not necessarily contain one hyperactive allotype or two hypoactive allotypes (96). Clearly, further studies are needed to better understand ERAP variant prevalence and functions in SpA.

One possible consequence of the presence of risk ERAP1 allotypes is that they exclusively produce arthritogenic peptides, while the other possible consequence is that they induce HLA-B^*^27 misfolding and expression of aberrant forms of HLA-B^*^27, resulting from abnormal peptide trimming in the ER, which causes a shortage of optimal peptides for HLA-B^*^27 loading. The current data on the effect of ERAP1 polymorphisms on the cell surface expression of misfolded HLA-B^*^27 are still controversial (97–99). This is at least in part due to the complex effects of polymorphic residues on the function of ERAP1. Predictions of the cleavage efficiencies of ERAP1 allotypes based on individual residue occurrences, frequently adopted in the literature, may not be fully accurate, and the effects of altered cleavage efficiencies could be variable for different HLA-B allotypes. In addition, the combined effects of ERAP1 and ERAP2 should be considered. ERAP1 and ERAP2 can form heterodimers to trim peptides with enhanced efficiency (100). Polymorphisms of ERAP2 also affect its activity (91) and thus the genotype of ERAP2 is an important consideration while studying the effect of ERAP1 on HLA-B^*^27 misfolding.

## Concluding Remarks

HLA-B^*^27 associated with the development of articular and extra-articular manifestations of SpA. Presence of HLA-B^*^27 also drives structural damage in the axial skeletal of individuals with axSpA. These clinical associations have brought to focus the role of HLA-B^*^27 in the pathogenesis of SpA. Since HLA-I molecules are highly polymorphic, the unique properties of HLA-B^*^27 have drawn much attention. Accumulating evidence implicates HLA-B^*^27 molecules in disease through a number of mechanisms, including presenting arthritogenic peptides, misfolding and induction of UPR, and producing aberrant conformers on the cell surface that engage innate immune receptors. However, the primary causal factor is yet to be identified. Misfolding and aberrant structural variants of HLA-B27 are linked to the IL-23/IL-17 axis, which was shown to be critical from GWAS studies and trials of novel biologic therapies. Recent advances indicate that SpA is caused by a combination of HLA-B^*^27 and other genetic factors. It is important to recognize that, although the association with HLA-B^*^27 is strong, various forms of SpA also occur in individuals carrying other HLA-B alleles. Studies of the common characteristics of HLA-B^*^27 with such allotypes might shed further light on the pathogenesis of SpA.

## Author Contributions

CK, JG, and MR designed the review structure. CK and JG wrote and edited the manuscript. MR, SB, and VN edited the manuscript. All authors contributed to the article and approved the submitted version.

## Conflict of Interest

The authors declare that the research was conducted in the absence of any commercial or financial relationships that could be construed as a potential conflict of interest.

## Abbreviations

ALK 2: Activin receptor-like kinase-2
ANKENT: Ankylosing enthesopathy
AS: Ankylosing Spondylitis
AxSpA: Axial Spondyloarthrits
BMP: Bone morphogenic protein
csDMARDs: Conventional synthetic diseases modifying anti-rheumatic drugs
DBA, Dilute: Brown and non-Agouti
ER: Endoplasmic reticulum
ERA: Enthesitis related arthritis
ERAP: Endoplasmic Reticulum Aminopeptidase
FDR: First degree relatives
FHC: Free heavy chain
GC: Glucocorticoids
HLA: Human leukocyte antigen
hβ2m: Human beta2-microglobulin
IBD: Inflammatory Bowel Diseases
IL: Interleukin
ILC: Innate-like lymphocyte
JAK: Janus Kinase
jPsA: Juvenile Psoriatic arthritis
jSpA: Juvenile Spondyloarthritis
KIR: Killer cell immunoglobulin-like receptor
LILR: Leukocyte immunoglobulin (Ig)-like receptors
MAIT: Mucosal-associated invariant T
MHC: Major histocompatibility complex
MSC: Mesenchymal stem cells
NPEPPS: Aminopeptidase Puromycin Sensitive
NSAIDs: Non-steroidal anti-inflammatory drugs
PBMCs: Peripheral blood mononuclear cells
PIR: Paired immunoglobulin-like receptors
PLC: Peptide-loading complex
PsA: Psoriatic arthritis
PSpA: Peripheral Spondyloarthritis
RA: Rheumatoid arthritis
RARB: Retinoic acid receptor-β
ReA: Reactive arthritis
SNP: Single-nucleotide polymorphism
SpA: Spondyloarthritis
TAP: Transporter associated with antigen processing
TCR: T cell receptor
TGF: Transforming growth factor
TNAP: Tissue-nonspecific alkaline phosphatase
TNF: Tumor necrosis factor
UPR: Unfolded protein response
XBP1: X-box–binding protein 1.
